# A novel CAVIN1/GAS5 axis suppresses skin fibrosis through mitochondrial fission-mediated attenuation of the TGF-β/Smad pathway

**DOI:** 10.1093/burnst/tkag052

**Published:** 2026-08-10

**Authors:** Hao Yang, Yuxi Zhou, Hailin Xu, Chengyan Huang, Shuting Li, Xiaohui Li, Xue Wang, Yongfei Chen, Peng Wang, Honglin Wu, Julin Xie, Jiayuan Zhu, Zhicheng Hu

**Affiliations:** Department of Burn and Wound Repair, First Affiliated Hospital of Sun Yat-sen University, 58 Zhongshan Er Road, Yuexiu District, Guangzhou 510080, China; Department of Burn and Wound Repair, First Affiliated Hospital of Sun Yat-sen University, 58 Zhongshan Er Road, Yuexiu District, Guangzhou 510080, China; Department of Dermatology, Dermatology Hospital of Southern Medical University, 2 Lujing Road, Yuexiu District, Guangzhou 510091, China; Department of Radiology, Zhujiang Hospital, Southern Medical University, 253 Gongye Avenue, Haizhu District, Guangzhou 510282, China; Department of Plastic Surgery, First Affiliated Hospital of Sun Yat-sen University, 58 Zhongshan Er Road, Yuexiu District, Guangzhou 510080, China; Department of Plastic Surgery & Burn Surgery, Shenzhen Hospital, Southern Medical University, 1333 Cuijing Road, Longhua District, Shenzhen 518000, China; Department of Burn and Wound Repair, First Affiliated Hospital of Sun Yat-sen University, 58 Zhongshan Er Road, Yuexiu District, Guangzhou 510080, China; Department of Burn and Wound Repair, First Affiliated Hospital of Sun Yat-sen University, 58 Zhongshan Er Road, Yuexiu District, Guangzhou 510080, China; Department of Burn and Wound Repair, First Affiliated Hospital of Sun Yat-sen University, 58 Zhongshan Er Road, Yuexiu District, Guangzhou 510080, China; Department of Burn and Wound Repair, First Affiliated Hospital of Sun Yat-sen University, 58 Zhongshan Er Road, Yuexiu District, Guangzhou 510080, China; Department of Burn and Wound Repair, First Affiliated Hospital of Sun Yat-sen University, 58 Zhongshan Er Road, Yuexiu District, Guangzhou 510080, China; Department of Burn and Wound Repair, First Affiliated Hospital of Sun Yat-sen University, 58 Zhongshan Er Road, Yuexiu District, Guangzhou 510080, China; Department of Burn and Wound Repair, First Affiliated Hospital of Sun Yat-sen University, 58 Zhongshan Er Road, Yuexiu District, Guangzhou 510080, China

**Keywords:** CAVIN1, Long non-coding RNA GAS5, Mitochondrial fission, Skin fibrosis, Keloid

## Abstract

**Background:**

Keloids, which are characterized by excessive collagen deposition and fibroblast hyperactivation, present significant therapeutic challenges because of their high recurrence rates and incompletely understood pathogenesis. The transforming growth factor-β (TGF-β) pathway is a central driver, but its upstream regulators in the development of skin fibrosis remain elusive. In this study, we aimed to identify novel upstream modulators of skin fibrosis and to elucidate the underlying molecular mechanisms by which these modulators regulate TGF-β signaling in keloid pathogenesis.

**Methods:**

We integrated results of transcriptomic and proteomic analyses of patient-derived keloid and normal skin tissues. Functional validation was performed by using primary keloid fibroblasts (KFs) with gain- and loss-of-function approaches, RNA immunoprecipitation sequencing (RIP-seq), and actinomycin D chase assays. Two independent bleomycin-induced skin fibrosis mouse models were employed for in vivo validation.

**Results:**

Multiomics profiling revealed that caveolae-associated protein 1 (CAVIN1) was consistently downregulated in keloids. CAVIN1 overexpression in KFs suppressed fibrotic phenotypes, including migration, invasion, collagen contraction, and extracellular matrix protein expression. Mechanistically, CAVIN1 directly bound to and stabilized the mitochondrial long noncoding RNA GAS5. This CAVIN1/GAS5 axis inhibited Drp1/Fis1-mediated mitochondrial fission and subsequent reactive oxygen species (ROS) production, leading to the attenuation of the canonical TGF-β/Smad2/3 signaling pathway. Critically, GAS5 silencing abrogated the antifibrotic effects of CAVIN1. Furthermore, database screening revealed that the histone deacetylase inhibitor vorinostat was a CAVIN1-upregulating compound. Both AAV-mediated CAVIN1 overexpression and vorinostat treatment significantly ameliorated skin fibrosis in the mouse models.

**Conclusions:**

Our study reports a novel CAVIN1/GAS5 axis that concurrently targets mitochondrial fission and the TGF-β/Smad pathway to suppress skin fibrosis. These findings not only reveal a previously unrecognized mechanistic link in keloid pathogenesis but also identify CAVIN1 as a promising therapeutic target, with vorinostat as a potential repurposable drug for treating fibrotic skin disorders.

## Graphical Abstract

**Graphical Abstract:**
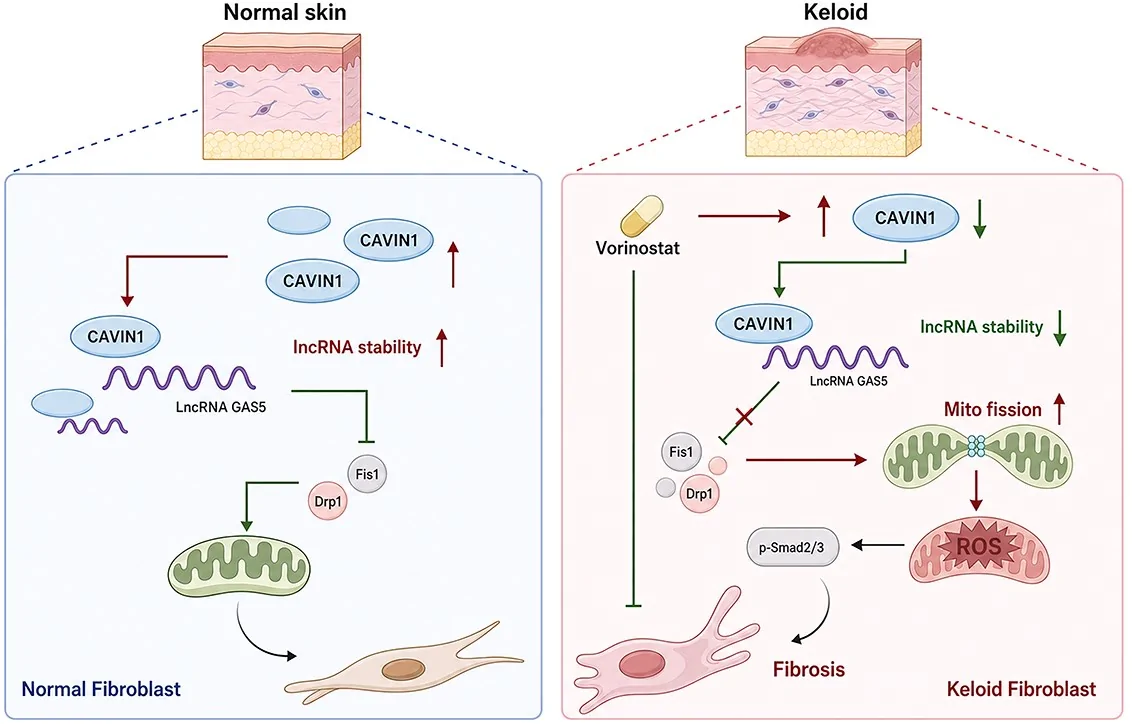

## Highlights

- Identifies CAVIN1 as a key downregulated factor in keloid pathogenesis.
- Elucidates a novel CAVIN1/GAS5 axis that directly binds and stabilizes mitochondrial long non-coding RNA GAS5, thereby suppressing Drp1/Fis1-mediated mitochondrial hyperfission, reducing reactive oxygen species production, and blocking TGF-β/Smad2/3 signaling to exert anti-fibrotic effects.
- Identifies Vorinostat as a compound capable of upregulating CAVIN1 expression and alleviating skin fibrosis in a mouse model, highlighting its potential for drug repurposing in fibrotic diseases.

## Background

Keloids are a common fibroproliferative skin disorder, and its core pathological features are abnormal hyperactivity of fibroblasts and excessive deposition of the extracellular matrix (ECM) collagen during aberrant wound healing [1]. Keloids not only impair patients’ motor function but also affect their quality of life and can even lead to psychological distress of varying severity [2, 3]. Currently, despite the availability of various treatment modalities, such as surgical excision, laser therapy, and intralesional corticosteroid injections, issues such as unstable efficacy and high recurrence rates are prevalent [4–6]. The fundamental reason lies in the fact that the pathogenesis of keloids has not yet been fully elucidated.

Currently, the transforming growth factor-β (TGF-β) signaling pathway is recognized as the core driver of the process of fibrosis [7]. In skin fibrosis, TGF-β signaling is persistently activated, leading to the phosphorylation of its downstream effector molecules, Smad2/3. Subsequently, phosphorylated Smad2/3 (p-Smad2/3) translocate into the nucleus, where they initiate the fibrotic program and directly upregulate gene expression of core ECM components, ultimately resulting in skin thickening and hardening [8]. Simultaneously, the recruitment of a large number of inflammatory cells to the wound area establishes a sustained low-grade inflammatory microenvironment, which in turn further stimulates TGF-β production, creating a vicious inflammation–fibrosis cycle that promotes continuous progression of fibrosis [9]. Consequently, targeting the TGF-β pathway remains a cornerstone of antifibrotic drug development. However, its upstream regulatory network is complex and not fully delineated, which necessitates the identification of novel master regulators.

Integrated multiomics approaches have emerged as powerful tools for systematically deciphering key disease modulators [10]. In this study, we combined the transcriptomic and proteomic profiles of patient-derived keloid and normal skin tissues. This strategy led to the identification of caveolae-associated protein 1 (CAVIN1), also known as polymerase I and transcript release factor (PTRF), as a key molecule that is consistently downregulated at both the mRNA and protein levels in keloids. CAVIN1 is a multifunctional protein involved in the formation and transcriptional regulation of caveolae [11, 12]. Notably, it has been reported to play a protective role in cardiac fibrosis [13]; however, its function and mechanism in skin fibrosis remain entirely unexplored.

We further discovered that CAVIN1 exerted its antifibrotic effect by posttranscriptionally regulating the long noncoding RNA GAS5. GAS5 is an lncRNA that is enriched in mitochondria and is known to modulate the cell cycle and apoptosis [14]. Notably, it has been shown to inhibit fibrosis in cardiac, hepatic, and renal contexts [15–17]. However, its role in skin fibrosis, particularly in relation to mitochondrial dynamics, is unknown. We hypothesized that CAVIN1 might stabilize GAS5 to influence keloid pathogenesis. Mechanistically, we identified a novel axis through which CAVIN1 binds to and stabilizes the GAS5 transcript. This action suppresses Drp1/Fis1-mediated mitochondrial fission and subsequent generation of reactive oxygen species (ROS), ultimately leading to the inhibition of the canonical TGF-β/Smad2/3 signaling pathway and the amelioration of fibrosis. Furthermore, by leveraging this mechanism, we screened the DSigDB and identified vorinostat, a histone deacetylase (HDAC) inhibitor [18], as a candidate drug that is capable of upregulating CAVIN1 expression.

In summary, this study employed a multiomics approach to identify CAVIN1 as a pivotal antifibrotic hub in keloids. We elucidated a previously unrecognized CAVIN1/GAS5 axis that was found to attenuate skin fibrosis by concurrently inhibiting mitochondrial fission and the TGF-β/Smad pathway. Our work not only provides novel insights into keloid pathogenesis but also highlights CAVIN1 as a promising therapeutic target, which is supported by the preclinical validation of vorinostat as a potential translational strategy for treating fibrotic skin disorders.

## Methods

### Clinical sample collection and analysis

The study sample set comprised keloid tissues from patients who underwent keloid surgery and healthy skin tissues from patients who underwent benign mass resection at the First Affiliated Hospital of Sun Yat-sen University, Guangdong Province, China. All keloid tissues were obtained from patients with clinically and histologically confirmed keloids who had not received any prior treatment for at least 6 months before surgery. Healthy skin tissues were collected from patients who underwent benign mass resection and who had no history of keloid formation or fibrotic disorders. To minimize confounding effects, we matched the keloid and control groups by age (±5 years), sex, and anatomical site (all samples were obtained from the chest or auricular region). Keloid samples were classified as active lesions on the basis of clinical criteria (progression beyond the original wound boundary, persistent pruritus or pain, and a duration <2 years). The tissue samples were transferred to the laboratory on ice within 30 minutes after surgical resection. Keloid tissues were harvested from the central region of the lesion, avoiding the margins and adjacent normal tissue. Normal skin tissues were collected from the incisional margin at least 2 cm away from the lesion border. Each tissue sample was divided into two portions; one was immediately snap-frozen in liquid nitrogen and stored at −80°C for subsequent RNA and protein extraction, and the other was fixed with 4% paraformaldehyde and embedded in paraffin for histological analysis. This study was approved by the Ethics Committee of the First Affiliated Hospital of Sun Yat-sen University (Approval No. 2021858), and each patient provided written informed consent.

Transcriptome and proteome analyses were conducted on the same sets of keloid (*n* = 3) and normal skin (*n* = 3) tissues with the assistance of Forevergen Biotechnology Co., Ltd (Guangzhou, China). RNA sequencing (RNA-seq) was performed by using the Illumina NovaSeq 6000 platform with a paired-end 150-bp read length. Proteomic analysis was conducted by using liquid chromatography–tandem mass spectrometry (LC–MS/MS) on a Q Exactive HF-X mass spectrometer (Thermo Fisher Scientific). Protein quantification was performed by using the label-free quantification (LFQ) algorithm in MaxQuant. Proteins with at least two unique peptides were retained for analysis. Genes with adjusted *P* values (false discovery rates, FDRs) ≤.001 and |log₂ fold changes| ≥ 1 were considered significantly differentially expressed genes (DEGs). Proteins with adjusted *P* values (FDRs) ≤.05 and |log₂ fold changes| ≥2 were considered significantly differentially expressed proteins (DEPs). The methods used for sequencing processing can be found in previous literature reports [19].

### Isolation and culture of fibroblasts

Primary keloid and normal human skin fibroblasts were isolated from keloid and normal skin samples, respectively, by using established protocols [20]. The tissues were thoroughly washed with PBS to remove the epidermis and subcutaneous adipose tissue and then cut into 1–3 mm^3^ pieces. Following digestion, the resulting suspension was filtered and centrifuged at 1500 rpm for 5 minutes. Both keloid and normal fibroblast cultures were maintained in a CO_2_ incubator. To investigate the effect of vorinostat on keloid fibroblasts (KFs), the cells were treated with vorinostat (MedChemExpress, USA) at a concentration of 0.5 μM for 48 hours.

### Cell transfection

For overexpression, full-length human CAVIN1 or GAS5 cDNA was cloned into the pcDNA3.1(+) vector to generate CAVIN1-OE and GAS5-OE plasmids. For knockdown, specific siRNAs against CAVIN1 (siCAVIN1) and GAS5 (siGAS5), along with a nontargeting control (siNC), were synthesized. All plasmids and siRNAs were synthesized and verified by Shanghai GeneChem Co., Ltd. Fibroblasts were transfected at 60%–70% confluence with the Lipofectamine 3000 reagent according to the manufacturer’s protocol. The cells were harvested 48 hours posttransfection for further analysis. Transcriptome analysis was assisted by Nobliobio Biotechnology Co., Ltd (Guangzhou, China). Total RNA was extracted from the samples, and ribosomal RNA was removed to enrich for lncRNAs and mRNAs. Strand-specific libraries were constructed and sequenced on the Illumina NovaSeq 6000 platform with a paired-end 150-bp read length. For differential expression analysis, genes or lncRNAs with a fold change ≥2 or ≤0.5 and a *P* value ≤.05 were considered significantly differentially expressed. RNA immunoprecipitation (RIP) experiments were performed by using the Magna RIP RNA-binding protein immunoprecipitation kit (No. 17–701; Millipore) according to the manufacturer’s instructions. For RIP-sequencing (RIP-seq), immunoprecipitated RNA libraries were constructed and sequenced with the technical support of Nobliobio Biotechnology Co., Ltd (Guangzhou, China). Sequencing was performed on the Illumina NovaSeq 6000 platform with a paired-end 150-bp read length. Peaks with a *q* value ≤0.05 (MACS2) were considered significant. For differential expression analysis, genes or lncRNAs with a fold change ≥2 or ≤0.5 and a *P* value ≤.05 were considered significantly differentially expressed. The methods used for sequencing processing can be found in previous literature reports [19]. To further validate the RIP-seq results, the RNA fraction precipitated by RIP was analyzed via quantitative PCR (RT–qPCR).

### Actinomycin D chase assay

Transcript stability was evaluated by using actinomycin D (S7418; Selleck). At 48 hours posttransfection, the cell culture medium was replaced with fresh complete medium containing 1 μM actinomycin D. Total RNA was then isolated from replicate wells at predetermined intervals (e.g. 0, 1, 2, 4, and 8 hours) after the addition of actinomycin D. The relative remaining levels of target mRNAs at each time point were determined by quantitative RT–PCR, and RNA decay rates were calculated accordingly.

### Western blot analysis

Protein was extracted from the cells and quantified with a BCA kit. A total of 30 μg of protein per sample was used for western blot analysis. The proteins were separated by 10% SDS–PAGE and subsequently transferred onto polyvinylidene fluoride (PVDF) membranes. Following blocking with 5% BSA at room temperature for 1 h, the blots were incubated overnight at 4°C with specific primary antibodies. On the following day, the blots were incubated with secondary antibodies for another hour. Finally, image acquisition was performed by using a MiniChemi chemiluminescence imager. The antibodies used are listed in Supplementary File 1.

### Reverse transcription–RT–qPCR

Total RNA was isolated from tissues with the TRIzol reagent (Invitrogen), followed by reverse transcription according to the manufacturer’s instructions (TaKaRa™) and real-time polymerase chain reaction with the TaKaRa SYBR Premix Ex Taq II (Perfect Real Time). GAPDH was used as an internal control, and the primer sequences are provided in Supplementary File 2. Relative gene expression levels were calculated by using the 2^−^ΔΔCt method, with the expression values normalized to those of the control group. For graphical presentation, the control group value was set to 1.

### Collagen gel contraction, migration and invasion assays

The KFs were combined with rat tail collagen (5 mg/ml) and 10× DMEM, followed by incubation in a 24-well plate at 37°C for 30 minutes. Subsequently, the resulting collagen gels were photographed after 24 hours. The difference in the gel areas was then calculated. Cell migration and invasion assays were conducted according to previously described methods. For quantification, five random fields per well were selected under magnification, and the number of stained cells was counted. Each experiment was independently repeated three times. The data are presented as the fold change relative to the control group, which was normalized to 1.

### Immunofluorescence

The cells were washed three times with PBS, fixed with 4% paraformaldehyde for 20 minutes, and permeabilized with 0.5% Triton X-100 for 20 minutes. After being blocked with 5% goat serum for 40 minutes, the cells were incubated overnight at 4°C with antibodies against collagens I and III, followed by incubation for 60 minutes at room temperature with Cy3- or CoraLite 488-conjugated secondary antibodies and nuclear counterstaining with DAPI. Finally, the samples were washed, mounted with an anti-fluorescence quencher, and observed under a fluorescence microscope. The antibodies used are listed in Supplementary File 1.

### Detection of ROS

After being washed with PBS, the treated cells were incubated with a DCFH-DA fluorescent probe (Beyotime, China) for 30 minutes at 37°C. After the incubation, the cells were washed to remove any unbound probe. The ROS fluorescence intensity was then measured via fluorescence microscopy.

### Animal models

All experimental protocols received approval from the First Affiliated Hospital of Sun Yat-sen University’s Animal Care and Use Committee (Approval No. 2021858). The first animal model was a bleomycin-induced dermal fibrosis model established with 6-week-old male C57BL/6 mice. Twenty-one days before fibrosis induction, the mice received a single subcutaneous injection (100 μl) of either AAV-CAVIN1 or AAV-control (AAV-CTR) (both at 1 × 10^12^ vg/ml) into the dorsal skin. The AAV vectors expressing mouse CAVIN1 (AAV-CAVIN1) or control (AAV-CTR) were constructed by using the pAAV-CMV-EGFP-3FLAG backbone (GeneChem, Shanghai, China). Fibrosis was then initiated by daily subcutaneous injection of 100 μl of bleomycin (0.5 mg/ml; MedChemExpress, USA) at the same site for four consecutive weeks. The preventive regimen was designed primarily to assess and demonstrate the protective role of CAVIN1 by evaluating whether overexpression of CAVIN1 could attenuate the initiation and progression of bleomycin-induced skin fibrosis.

The second animal model was a bleomycin-induced dermal fibrosis model established with 8-week-old male C57BL/6 mice. Fibrosis was first induced by daily subcutaneous injection of 100 μl of bleomycin (1 mg/ml) into the dorsal skin. Beginning on Day 16 after the initial bleomycin injection, the mice were randomly divided into a treatment group and a vehicle control group. The treatment group received subcutaneous injections of vorinostat (MedChemExpress, USA) at a concentration of 50 μM in a 100-μl volume, which was administered around the fibrotic lesion site every other day for a total of six doses. The vehicle control group received an equivalent volume of dimethyl sulfoxide (DMSO) on the same schedule. After the skin fibrosis model was established, therapeutic intervention was performed to determine whether vorinostat could reverse established fibrosis, thereby modeling a clinically relevant treatment scenario.

All the mice were sacrificed 4 weeks after the start of the bleomycin injections. Dorsal skin samples were harvested for subsequent analysis, including RT–qPCR, hematoxylin and eosin (H&E) staining, Sirius Red staining, and Masson’s trichrome staining.

### Immunohistochemical and histological analyses

Skin samples were paraffin-embedded and sectioned into 4-μm sections, followed by H&E, Masson’s trichrome, or Sirius Red staining. For immunohistochemical staining, tissue sections from patients were deparaffinized with xylene, and antigen retrieval was performed in 10 mM sodium citrate buffer (pH 8.0) via full-power pressure cooking for 5 minutes. The sections were then treated with 3% hydrogen peroxide at room temperature for 25 minutes to inhibit endogenous peroxidase activity, after which endogenous antigens were blocked by using 3% bovine serum albumin (BSA). The sections were subsequently incubated with both primary and secondary antibodies. The antibodies used are listed in Supplementary File 2. The sections were visualized with a 3,3′-diaminobenzidine (DAB) chromogenic solution and counterstained with hematoxylin before they were mounted.

### Statistical analysis

Data analysis and figure generation were conducted by using GraphPad Prism version 8.0 (GraphPad Software, San Diego, CA, USA). The results are presented as the mean ± standard deviation (SD) from at least three independent experiments, unless otherwise specified. The normality of the data distribution was assessed with the Shapiro–Wilk test. For normally distributed data, comparisons between two groups were performed by using two-tailed unpaired Student’s *t* tests, and comparisons among more than two groups were performed by using one-way analysis of variance (ANOVA), followed by Tukey’s post hoc test for multiple comparisons to adjust for the familywise error rate. For data that did not pass the normality test, nonparametric tests were applied, including the Mann–Whitney *U* test for two-group comparisons and the Kruskal–Wallis test, followed by Dunn’s post hoc test, for multiple-group comparisons. For the two-factor design, data were analyzed using two-way ANOVA with Šídák’s multiple comparisons test. In all the analyses, a *P* value <0.05 was considered to indicate statistical significance. No statistical methods were used to predetermine the sample size; however, sample sizes were chosen on the basis of the conventions in the field and preliminary experiments to ensure adequate power to detect biologically meaningful differences. For in vitro experiments, each independent biological replicate consisted of cells from a distinct donor or passage. For in vivo experiments, group sizes are indicated in the figure legends.

## Results

### Identification of CAVIN1 as a key target in keloid pathogenesis

**Figure 1:**
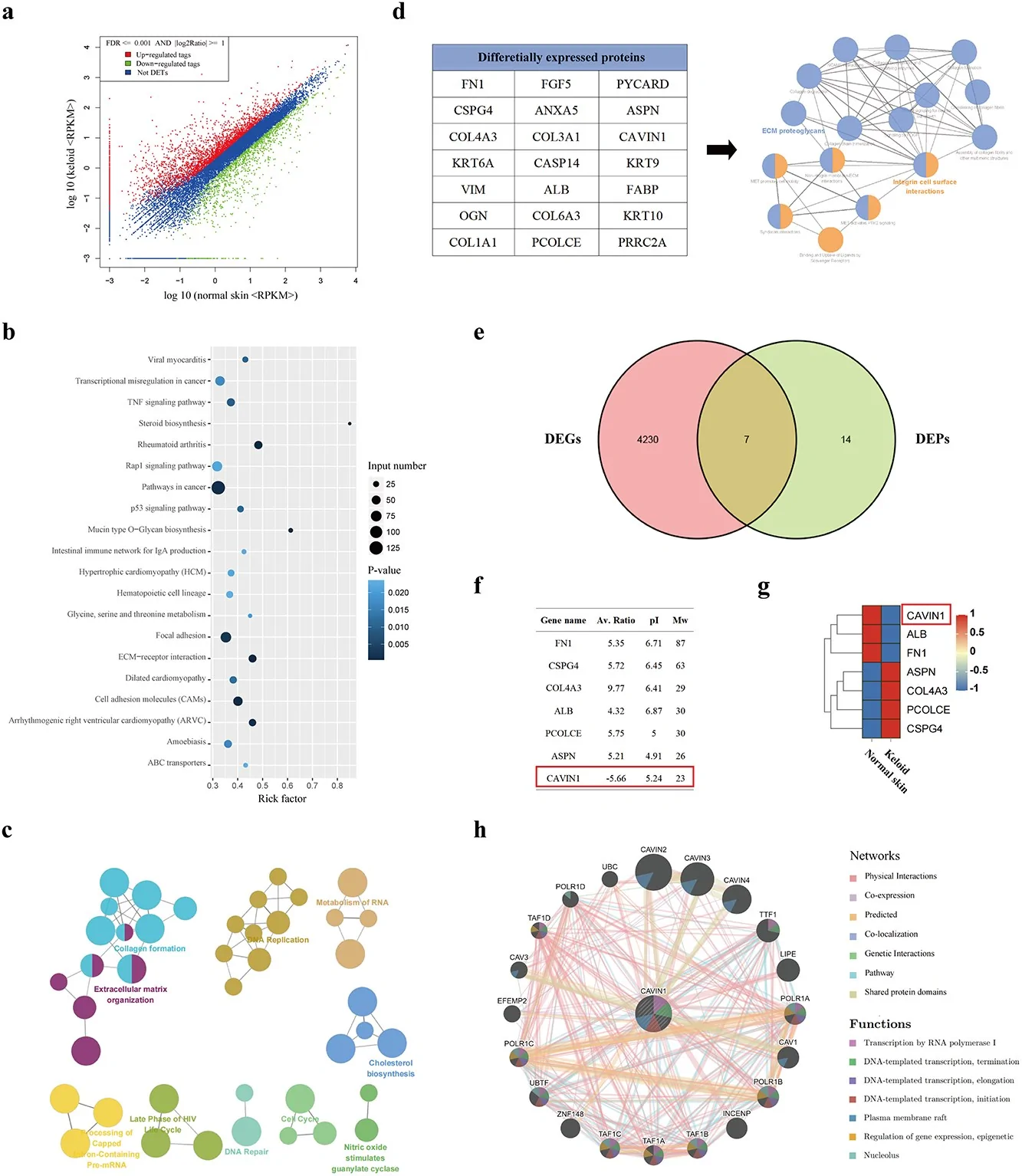
Identification of CAVIN1 as a key target in keloid pathogenesis. (**a**) DEGs between keloid and normal skin among the total genes. Each tag represents a single gene. Red tags indicate upregulated genes, green tags indicate downregulated genes, and blue tags indicate genes that were not differentially expressed. (**b**) Results of the KEGG pathway enrichment analysis of the DEGs. (**c**) Network modules depicting enriched biological processes of the DEGs. (**d**) DEPs between keloid and normal skin and their associated biological process network. (**e**) Venn diagram showing the overlap between the screened DEGs and DEPs. (**f**) Results of the differential analysis of overlapping DEPs. (**g**) Heatmap of overlapping DEGs. The data are presented as the mean expression values for each group. The color scale represents Z score-normalized expression levels (blue: low expression; red: high expression). (**h**) Molecular interaction network of CAVIN1 and associated functional annotations. Nodes are color coded by function type, and edges are distinguished by color to represent different interaction categories. *DEGs* differentially expressed proteins, *CAVIN1* caveolae-associated protein 1

To identify key molecules involved in keloid pathogenesis, we first performed a transcriptomic analysis on patient-derived keloid tissues and healthy skin controls. A total of 4237 significant DEGs were identified (Figure 1a). Kyoto Encyclopedia of Genes and Genomes (KEGG) pathway enrichment analysis revealed that these DEGs were prominently enriched in the ECM–receptor interaction pathway (Figure 1b). A GO term clustering network that was constructed from the enriched terms demonstrated a strong functional synergy between ‘collagen formation’ and ‘extracellular matrix organization’ (Figure 1c). Proteomic profiling of the same tissue sets revealed 21 significant DEPs, from which key molecules were selected to construct a GO pathway clustering network that displayed notable similarity to the transcriptomic enrichment profile (Figure 1d). Integration of the transcriptomic and proteomic datasets via Venn diagram analysis revealed 7 overlapping molecules (Figure 1e). Among these molecules, CAVIN1 was uniquely downregulated at the protein level in keloids, a finding consistent with its transcriptional downregulation, as visualized in a heatmap displaying the mean expression values for each group (Figure 1f, g). Molecular interaction network analysis revealed that CAVIN1 interacted with multiple signaling factors and was primarily associated with gene regulation (Figure 1h). Collectively, our multiomics data reveal that CAVIN1 is significantly downregulated during keloid pathogenesis, which suggests that it may exert regulatory effects through the modulation of fibrosis-related pathways.

### CAVIN1 attenuates the fibrotic phenotype by inhibiting fibroblast activation

**Figure 2:**
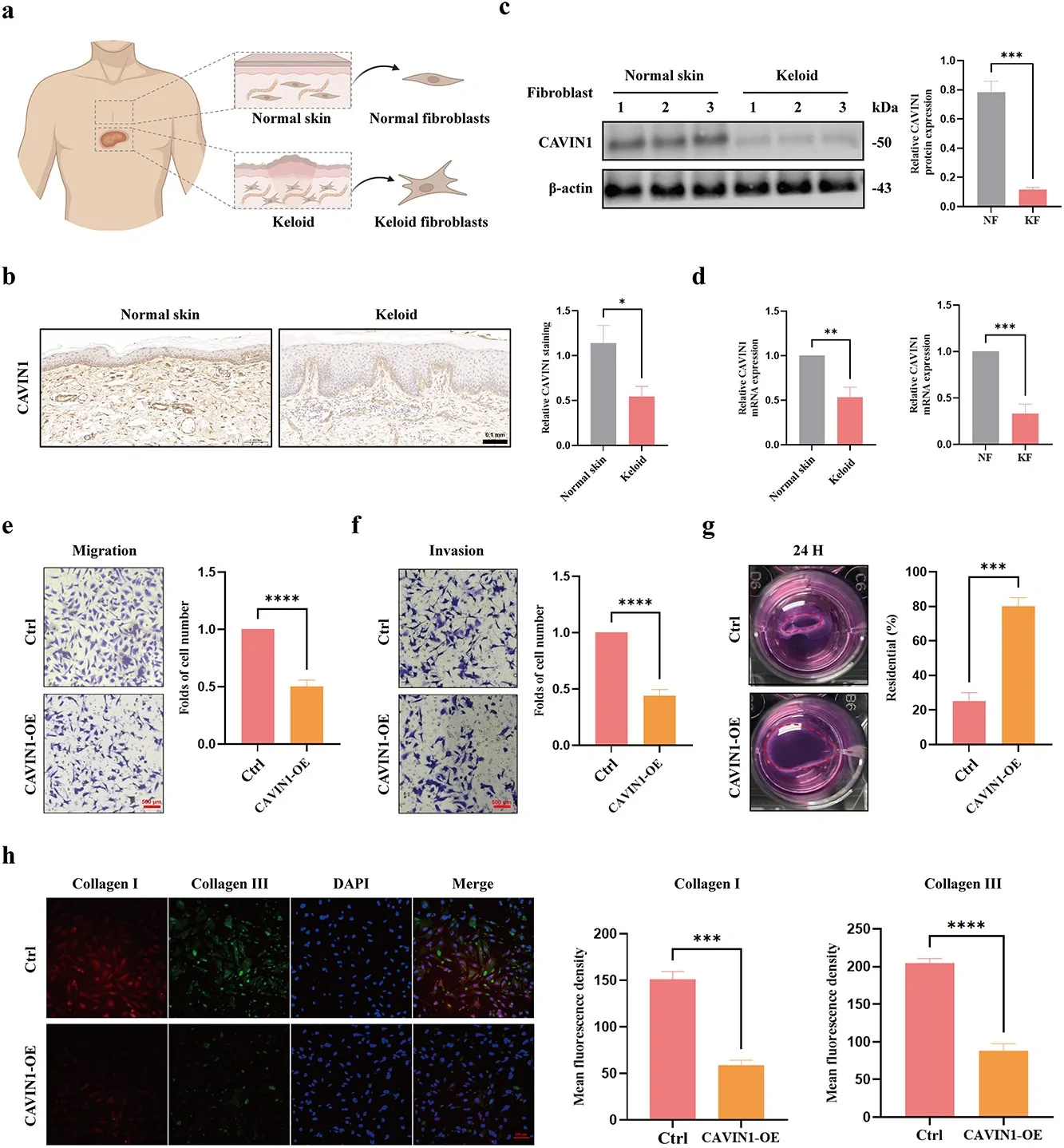
CAVIN1 attenuates the fibrotic phenotype by inhibiting fibroblast activation. (**a**) Schematic diagram of fibroblast isolation from normal skin and keloid tissues. (**b**) Representative images and quantification results of immunohistochemical staining for CAVIN1 in tissue sections (*n* = 3). Scale bar: 0.1 mm. (**c**) Results of the western blot analysis and quantification of CAVIN1 expression in NFs versus KFs (*n* = 3). (**d**) Results of the quantitative analysis of CAVIN1 mRNA levels in tissues and cultured fibroblasts from normal skin and keloids (*n* = 3). (**e**, **f**) representative images and quantification results of the cell migration and invasion assays in CTR and CAVIN1-OE KFs (*n* = 3). Scale bar: 500 μm. (**g**) Representative images and quantification results of the collagen gel contraction assays at 24 hours posttreatment in CTR and CAVIN1-OE KFs (*n* = 3). The dashed lines outline the gel boundaries. (**h**) Representative IF images and quantification results of collagen I (red) and collagen III (green) costaining in in vitro-cultured CTR and CAVIN1-OE KFs (*n* = 3). Nuclei were counterstained with DAPI (blue). Scale bar: 100 μm. ^*^P <0.05, ^**^P <0.01, ^***^P <0.005, ^****^P <0.001. NFs normal skin fibroblasts, *KFs* keloid fibroblasts, *Ctrl* control *CAVIN1* caveolae-associated protein 1, *CAVIN1-OE* CAVIN1-overexpressing

Normal skin and keloid tissues were obtained from the enrolled patients, and corresponding fibroblasts were subsequently isolated from these tissues (Figure 2a). Immunohistochemical staining confirmed a marked decrease in CAVIN1 expression in keloid tissues compared with normal skin (Figure 2b). Consistent with these findings, western blot analysis revealed significantly lower CAVIN1 protein levels in KFs than in normal skin fibroblasts (NFs) (Figure 2c). Furthermore, RT–qPCR analysis demonstrated significant downregulation of CAVIN1 mRNA in both keloid tissues and KFs compared with their respective controls (Figure 2d). To investigate the functional impact of CAVIN1, we generated CAVIN1-overexpressing (CAVIN1-OE) KFs. Compared with control cells, CAVIN1-OE KFs exhibited significantly suppressed migration and invasion (Figure 2e and f). In addition, collagen gel contraction assays revealed that, compared with that in the control group, the contractile capacity of fibroblasts was inhibited in the CAVIN1 overexpression group (Figure 2g). Immunofluorescence (IF) staining of cultured KFs further revealed that CAVIN1 overexpression led to a marked decrease in the levels of both collagen I and collagen III (Figure 2h), suggesting that CAVIN1 may alleviate fibrosis by suppressing collagen production. Collectively, these results indicate that CAVIN1 is downregulated in keloids and that its overexpression can attenuate skin fibrosis by inhibiting key fibroblast functions and reducing collagen deposition.

### Regulatory mechanism of CAVIN1 overexpression in KFs

**Figure 3:**
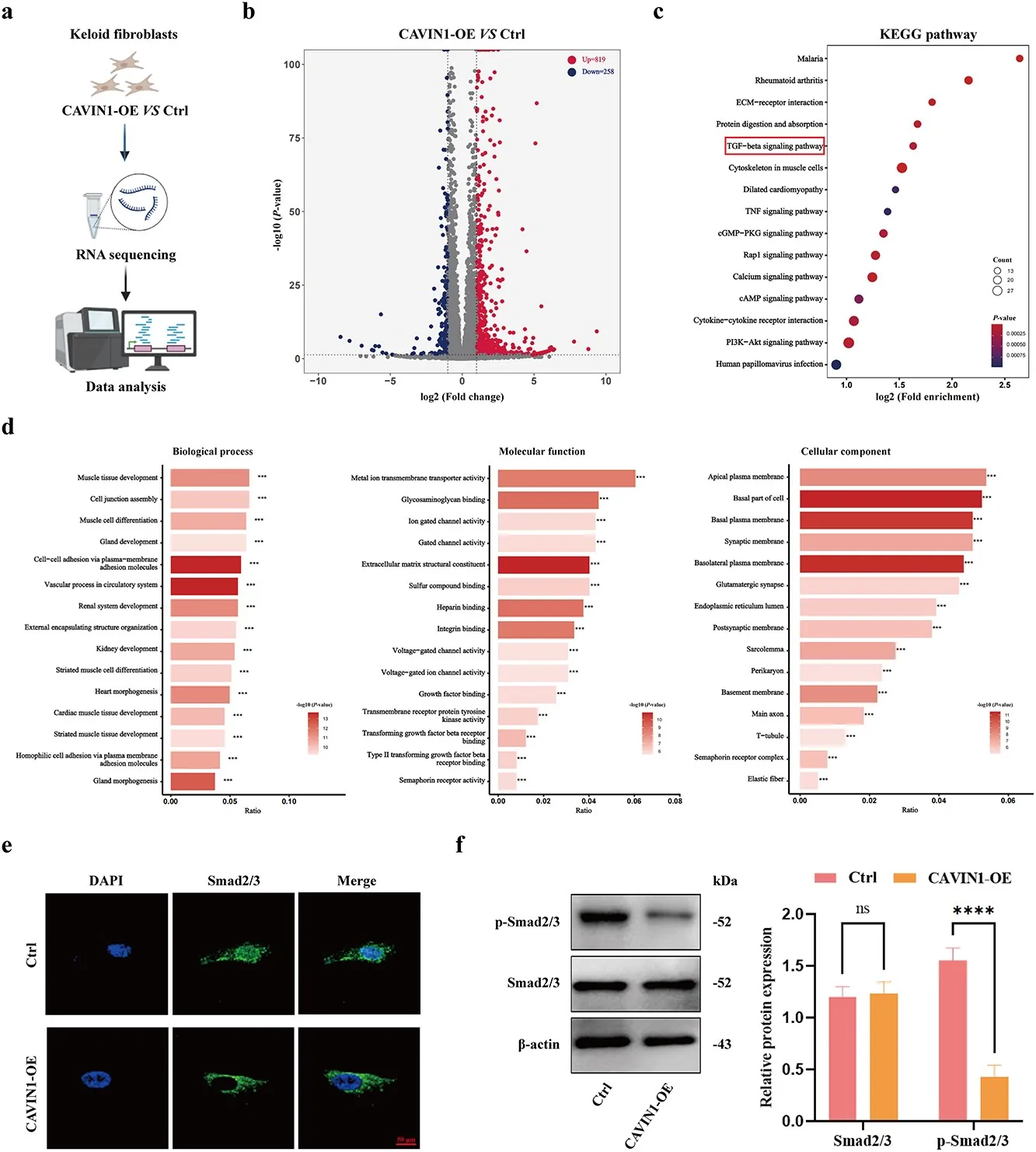
Regulatory mechanism of CAVIN1 overexpression in KFs. (**a**) Schematic workflow of the RNA-seq analysis performed in KFs. (**b**) Volcano plot displaying DEGs between CAVIN1-OE and control KFs. (**c**) Results of the KEGG pathway enrichment analysis of genes that were significantly upregulated upon CAVIN1 overexpression. (**d**) Results of the functional enrichment analysis based on GO terms, including BP, MF, and CC categories. (**e**) Representative IF images of Smad2/3 (green) expression in CTR and CAVIN1-OE KFs. Nuclei were counterstained with DAPI (blue). Scale bar: 50 μm. (**f**) Results of the western blot analysis and corresponding quantification of total Smad2/3 and p-Smad2/3 protein levels in the indicated groups (*n* = 3). ^****^P < 0.001; *ns* not significant. *KFs* keloid fibroblasts,*CAVIN1* caveolae-associated protein 1, *CAVIN1-OE* CAVIN1-overexpressing, *Ctrl* control, *BP* biological process, *MF* molecular function, *CC* cellular component

To elucidate the mechanism by which CAVIN1 attenuates the fibrotic phenotype, we performed RNA-seq on CAVIN1-OE and control KFs (Figure 3a). A total of 1077 DEGs were identified, comprising 819 upregulated and 258 downregulated genes (Figure 3b). KEGG enrichment analysis of the upregulated DEGs revealed significant enrichment of the TGF-β signaling pathway (Figure 3c), a central driver of fibrosis, which is known to promote fibroblast activation and ECM deposition. Consistent with these findings, the results of the GO enrichment analysis highlighted related biological processes and molecular functions, including “cell–cell adhesion via plasma-membrane adhesion molecules,” “homophilic cell adhesion via plasma-membrane adhesion molecules”, “transforming growth factor beta receptor binding”, and “extracellular matrix structural constituent” (Figure 3d). Given the pivotal role of the canonical TGF-β/Smad pathway in fibrogenesis, we next investigated whether CAVIN1 overexpression modulates this signaling cascade. IF staining demonstrated that CAVIN1 overexpression markedly reduced the nuclear accumulation of Smad2/3 (Figure 3e), indicating impaired transcriptional activity of this pathway. These observations were further corroborated by the data of western blot analysis, which revealed that CAVIN1 overexpression significantly suppressed the phosphorylation of Smad2/3 (Figure 3f). Collectively, these results demonstrate that CAVIN1 attenuates skin fibrosis by inhibiting the TGF-β/Smad2/3 signaling pathway in KFs. However, the upstream molecular mechanism through which CAVIN1 represses this transcriptional activation remains unknown. We therefore sought to identify direct targets of CAVIN1 that could mediate its antifibrotic effects.

### CAVIN1 maintains the mRNA stability of the lncRNA GAS5

**Figure 4:**
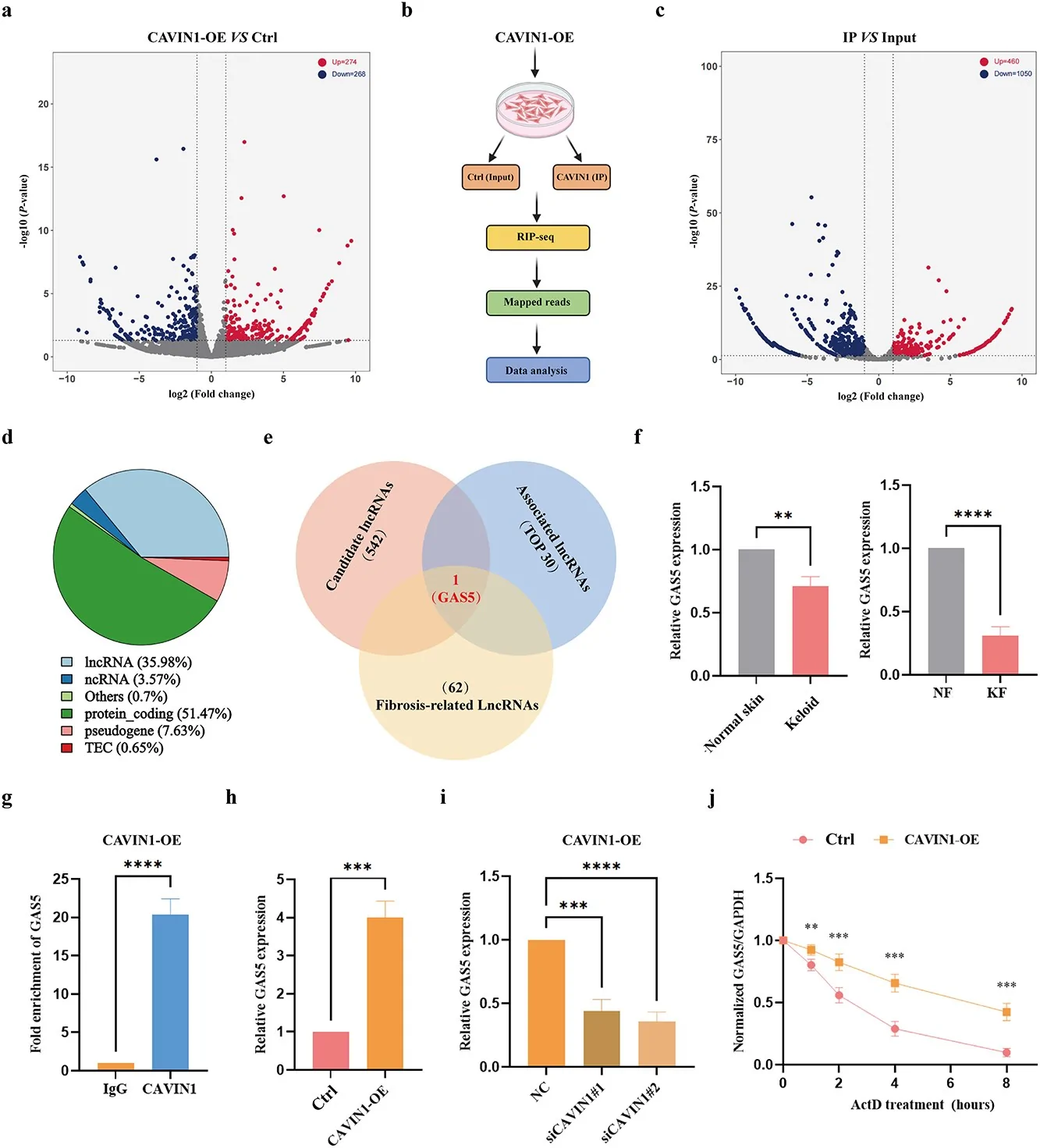
CAVIN1 maintains the mRNA stability of the lncRNA GAS5. (**a**) Volcano plot displaying lncRNAs that were differentially expressed between CAVIN1-OE and CTR KFs. (**b**) Schematic workflow of the RIP-seq analysis, in CAVIN1-OE KFs. (**c**) Volcano plot of RNAs that were significantly enriched in the CAVIN1 IP group compared with the input control group. (**d**) Pie chart showing the distribution of RNA categories identified on the basis of the CAVIN1 RIP-seq data. (**e**) Venn diagram showing the overlap among (i) differentially expressed lncRNAs according to the RNA-seq data, (ii) the top 30 lncRNAs bound to CAVIN1 according to the RIP-seq data, and (iii) 62 fibrosis-associated lncRNAs from a public database. The lncRNA GAS5 was identified as a common candidate. (**f**) Results of the quantitative analysis of GAS5 expression levels in normal skin and keloid tissues and cultured fibroblasts (*n* = 3). (**g**) RIP–qPCR validation of GAS5 enrichment by using a CAVIN1-specific antibody in CAVIN1-OE KFs. (**h**) Relative GAS5 expression levels in CTR and CAVIN1-OE KFs (*n* = 3). (**i**) Relative GAS5 expression levels in CAVIN1-OE KFs following transfection with siCAVIN1 (*n* = 3). (**j**) Results of the RT–qPCR analysis of the expression of GAS5 in CTR and CAVIN1-OE KFs treated with ActD at the indicated time points (*n* = 3). ^**^P <0.01, ^***^P < 0.005, ^****^P < 0.001. *CAVIN1* caveolae-associated protein 1, *CAVIN1-OE* CAVIN1-overexpressing, *Ctrl* control, *NFs* normal skin fibroblasts, *KFs* keloid fibroblasts, *IP* immunoprecipitation

Given that CAVIN1 is involved in gene transcription regulation and can function as an RNA-binding protein, we hypothesized that it might exert its antifibrotic effects by posttranscriptionally modulating specific RNA targets [21]. To test this hypothesis, we first identified differentially expressed long noncoding RNAs (lncRNAs) between CAVIN1-OE and control KFs (Figure 4a). To elucidate potential RNA targets of CAVIN1, we then performed RNA-binding protein immunoprecipitation, followed by RIP-seq, with an anti-CAVIN1 antibody to identify RNAs that directly interacted with CAVIN1 (Figure 4b). Analysis of the immunoprecipitates revealed a specific set of RNAs that were significantly enriched in the CAVIN1-IP samples compared with the IgG control samples (Figure 4c); the compositional profile of the RNAs is detailed in Figure 4d. To identify candidate RNAs, we performed an intersection analysis of the following three distinct RNA sets: (i) differentially expressed lncRNAs; (ii) a curated list of the top 30 CAVIN1-associated lncRNAs; and (iii) fibrosis-linked lncRNAs from the Fibrotic Disease-associated RNAome database (FDRdb). This integrated approach led to the identification of the lncRNA GAS5 as the sole overlapping candidate (Figure 4e). Quantitative analysis revealed that compared with their normal counterparts, both keloid tissues and KFs exhibited significant downregulation of GAS5 (Figure 4f), in parallel with the downregulation of CAVIN1 itself. To confirm the direct interaction between CAVIN1 and GAS5, as suggested by RIP-seq, we performed RIP-qPCR, which revealed a specific enrichment of GAS5 in the CAVIN1 immunoprecipitates relative to that in the IgG control (Figure 4g). We then investigated whether CAVIN1 regulates GAS5 expression. RT–qPCR analysis revealed that compared with those in control KFs, GAS5 mRNA levels in CAVIN1-OE KFs significantly increased (Figure 4h). Conversely, knocking down CAVIN1 in the GAS5 overexpression background effectively reduced GAS5 expression (Figure 4i). To determine whether this regulation occurs at the posttranscriptional level, we assessed GAS5 mRNA stability by using actinomycin D to inhibit new RNA synthesis. Decay curve analysis (0–8 h) revealed that GAS5 mRNA degradation was significantly slower in CAVIN1-OE KFs than in control KFs, demonstrating that CAVIN1 improves the transcriptional stability of GAS5 (Figure 4j). Given that GAS5 is a mitochondrion-localized lncRNA and is implicated in fibrosis in other contexts, we next investigated whether it mediates the antifibrotic function of CAVIN1 by regulating mitochondrial dynamics in KFs.

### Targeting the CAVIN1/GAS5 axis attenuates fibrosis by inhibiting mitochondrial fission

**Figure 5:**
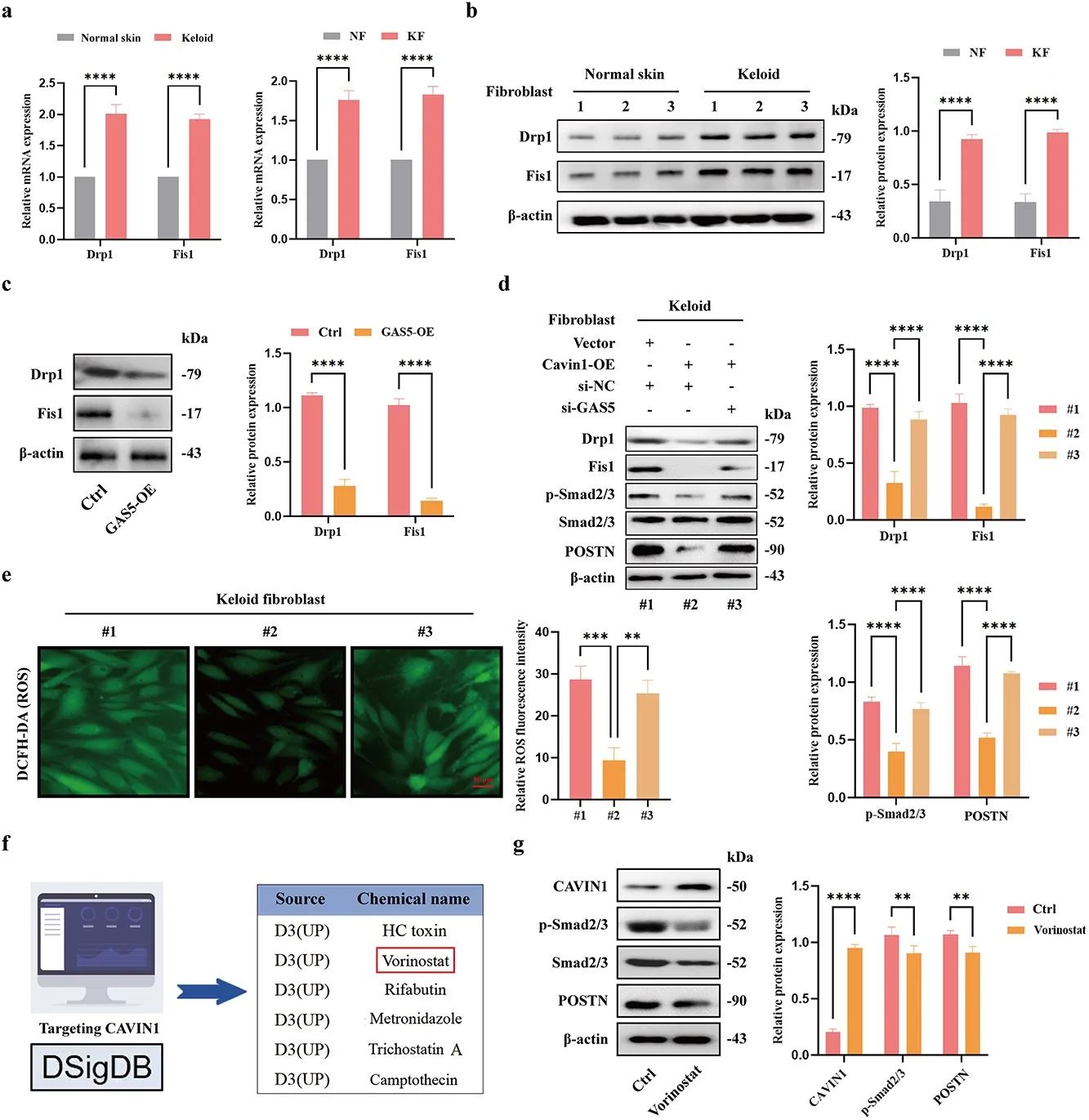
Targeting the CAVIN1/GAS5 axis attenuates fibrosis by inhibiting mitochondrial fission. (**a**) Results of the quantitative analysis of Drp1 and Fis1 mRNA levels in tissues and cultured fibroblasts from normal skin and keloids (*n* = 3). (**b**) Results of the western blot analysis and quantification of Drp1 and Fis1 protein expression levels in NFs versus KFs (*n* = 3). (**c**) Results of the western blot analysis and quantification of Drp1 and Fis1 protein expression levels in CTR and GAS5-OE KFs (*n* = 3). (**d**) Results of the western blot analysis and quantification of the effects of the vector, CAVIN1-OE, siNC, and siGAS5 on the protein levels of Drp1, Fis1, p-Smad2/3, and Postn in KFs (*n* = 3). (**e**) Representative images and quantification results of intracellular ROS levels detected by DCFH-DA staining in the indicated groups of KFs (*n* = 3). Scale bar: 50 μm. (**f**) Screening results from the DSigDB to identify candidate drugs targeting CAVIN1. (**g**) Results of the western blot analysis and quantification of CAVIN1, p-Smad2/3, and Postn levels in vorinostat-treated KFs (*n* = 3). ^**^P <0.01, ^***^P < 0.005, ^****^P < 0.001. *CAVIN1* caveolae-associated protein 1, *NFs* normal skin fibroblasts, *KFs* keloid fibroblasts, *Ctrl* control, *ROS* reactive oxygen species

The lncRNA GAS5 is localized to mitochondria and has been reported to attenuate fibrosis in other organ systems by regulating mitochondrial dynamics; however, its role and the involvement of mitochondrial fission in skin fibrosis remain unknown [15]. To address this gap, we first examined markers of mitochondrial fission. Compared with those in normal controls, both the mRNA and protein levels of the key fission regulators Drp1 and Fis1 were significantly elevated in keloid tissues and KFs (Figure 5a and b), indicating the aberrant activation of mitochondrial fission during keloid pathogenesis.

To directly investigate the role of GAS5 in this process, we overexpressed GAS5 in KFs and observed significant downregulation of both Drp1 and Fis1 expression (Figure 5c), confirming that GAS5 suppresses mitochondrial fission. We next investigated whether CAVIN1 inhibits this fission pathway and the fibrotic TGF-β/Smad axis in a GAS5-dependent manner. Western blot analysis revealed that CAVIN1 overexpression in KFs reduced the protein levels of Drp1 and Fis1 while concurrently suppressing Smad2/3 phosphorylation and the expression of the profibrotic marker periostin (POSTN) (Figure 5d). Critically, GAS5 silencing completely abrogated these suppressive effects, indicating that GAS5 is an indispensable downstream effector that mediates the concurrent inhibition of mitochondrial fission and the TGF-β/Smad pathway by CAVIN1. Considering that the overexpression of GAS5 alone was sufficient to suppress mitochondrial fission, these findings demonstrate that GAS5 is both necessary for the antifibrotic effects of CAVIN1 and sufficient to regulate the fission machinery. Given the established link between excessive mitochondrial fission and elevated ROS levels [22], we performed ROS staining. The results showed that CAVIN1-overexpressing fibroblasts exhibited significantly reduced ROS levels, an effect that was abolished by GAS5 knockdown (Figure 5e). These results indicate that CAVIN1 attenuates ROS production by inhibiting Drp1/Fis1-mediated mitochondrial fission via GAS5. As ROS directly activate the TGF-β/Smad signaling pathway, these findings collectively delineate a mechanistic cascade in which the CAVIN1/GAS5 axis suppresses Drp1/Fis1-mediated mitochondrial fission, reduces ROS generation, and subsequently inhibits TGF-β/Smad activation and fibrotic gene expression.

On the basis of the central role of CAVIN1 in this antifibrotic pathway, we explored its therapeutic potential by screening the DSigDB, which led to the identification of vorinostat, an established HDAC inhibitor, as a candidate drug predicted to target CAVIN1 (Figure 5f). Treatment of fibroblasts with vorinostat significantly upregulated CAVIN1 expression and concurrently inhibited the activity of the TGF-β/Smad2/3/Postn pathway (Figure 5g), supporting the potential of vorinostat as a CAVIN1-modulating agent. In summary, our findings reveal a pathway in which CAVIN1 stabilizes GAS5 to inhibit mitochondrial fission and ROS generation, thereby suppressing the TGF-β/Smad signaling axis and exerting an antifibrotic effect. Furthermore, the identification of vorinostat as a CAVIN1-upregulating compound highlights a promising translational strategy for keloid therapy.

### CAVIN1 is a druggable target for ameliorating skin fibrosis in vivo

**Figure 6:**
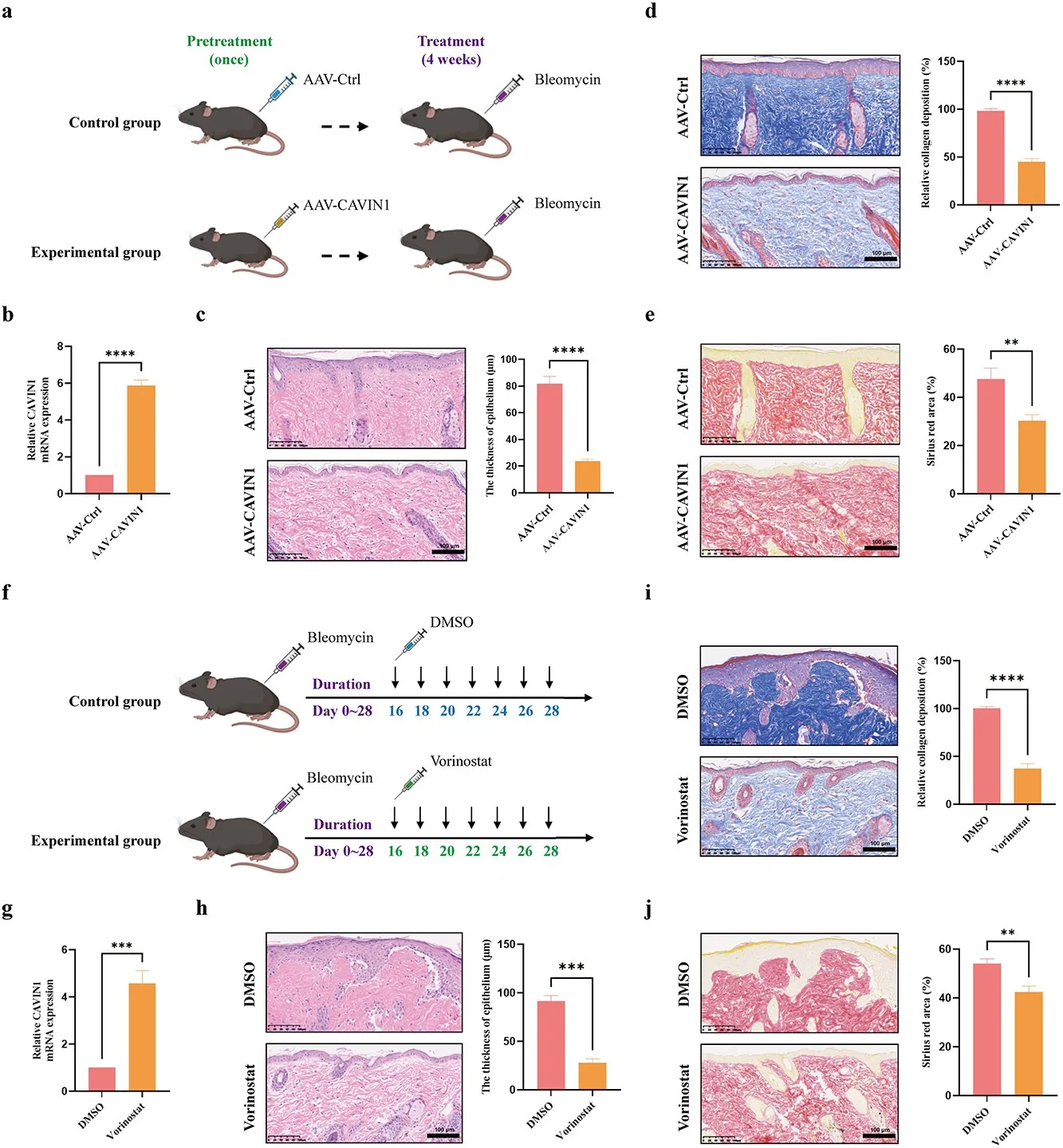
CAVIN1 is a druggable target for ameliorating skin fibrosis *in vivo*. (**a**) Schematic of the experimental design for the mouse skin fibrosis model and AAV-mediated intervention. (**b**) Results of the quantitative analysis of CAVIN1 mRNA expression in skin tissues from the indicated groups (*n* = 3). (**c**) Representative H&E-stained sections and quantification results of epidermal thickness in mice treated with the AAV-CTR or AAV-CAVIN1 (*n* = 3). Scale bar: 100 μm. (**d**) Representative Masson’s trichrome-stained sections (collagen in blue) and quantification results of collagen deposition in the AAV-CTR and AAV-CAVIN1 groups (*n* = 3). Scale bar: 100 μm. (**e**) Representative Sirius red-stained images and quantification results of collagen fibers in the AAV-CTR and AAV-CAVIN1 groups (*n* = 3). Scale bar: 100 μm. (**f**) Schematic of the mouse fibrosis model and the drug intervention regimen with vorinostat. (**g**) Results of the quantitative analysis of CAVIN1 mRNA expression in skin tissues after vorinostat treatment (*n* = 3). (**h**) Representative H&E-stained sections and quantification results of epidermal thickness in the vehicle (DMSO)- and vorinostat-treated groups (*n* = 3). Scale bar: 100 μm. (**i**) Representative Masson’s trichrome-stained sections and quantification results of collagen deposition in the DMSO and vorinostat groups (*n* = 3). Scale bar: 100 μm. (**j**) Representative Picrosirius red-stained sections and quantification results of collagen fibers in the DMSO and vorinostat groups (*n* = 3). Scale bar: 100 μm. ^**^P <0.01, ^***^P <0.005, ^****^P <0.001. *CAVIN1* caveolae-associated protein 1, *AAV* adeno-associated virus, *Ctrl* control

To validate the antifibrotic potential of targeting CAVIN1 in vivo, we performed two independent animal experiments. In the first experiment, mice were pretreated with either a control AAV (AAV-CTR) or an AAV overexpressing CAVIN1 (AAV-CAVIN1), followed by injection of bleomycin to induce skin fibrosis (Figure 6a). RT–qPCR confirmed the significant upregulation of CAVIN1 expression in skin tissues from the AAV-CAVIN1 group compared with those from the control group (Figure 6b). Western blot analysis revealed that, compared with the control group, skin tissues from the AAV-CAVIN1 group exhibited significantly upregulated expression of CAVIN1 protein, accompanied by decreased expression of Drp1 and Fis1 as well as reduced phosphorylation of Smad2/3 (Figure S1a). RT-qPCR further confirmed that GAS5 expression was significantly upregulated at the RNA level in the AAV-CAVIN1 group compared with the control group (Figure S1b). Histological analyses revealed that CAVIN1 overexpression substantially ameliorated fibrotic pathology. Thus, H&E staining revealed reduced epidermal thickening (Figure 6c), Masson’s trichrome staining indicated decreased collagen deposition (Figure 6d), and Sirius Red staining further confirmed a lower percentage of the collagen area (Figure 6e). In a second experiment, which included therapeutic intervention, a bleomycin-induced fibrosis model was first established. Beginning on Day 16, the mice received subcutaneous injections of either vehicle (DMSO) or the CAVIN1-inducing agent vorinostat (Figure 6f) every other day. RT–qPCR analysis demonstrated that vorinostat treatment successfully upregulated CAVIN1 expression (Figure 6g). Western blot analysis confirmed that vorinostat treatment increased CAVIN1 protein expression, accompanied by decreased Drp1, Fis1, and p-Smad2/3 levels **(**Figure S1c**)**. In addition, RT-qPCR demonstrated that GAS5 RNA expression was also significantly upregulated following vorinostat treatment **(**Figure S1d**)**. Consistent with the gene overexpression approach, vorinostat administration resulted in a thinner epidermis (Figure 6h), less collagen accumulation (Figure 6i), and a reduced collagen area (Figure 6j). Together, these results support that CAVIN1-mediated GAS5 stabilization inhibits mitochondrial fission and SMAD pathway activation, demonstrating that both genetic upregulation and pharmacological activation of CAVIN1 effectively attenuate skin fibrosis in vivo. These findings not only validate CAVIN1 as a promising therapeutic target but also provide preclinical evidence supporting the translational potential of vorinostat, a drug that was confirmed to act upon this newly characterized CAVIN1/GAS5 axis.

## Discussion

**Figure 7:**
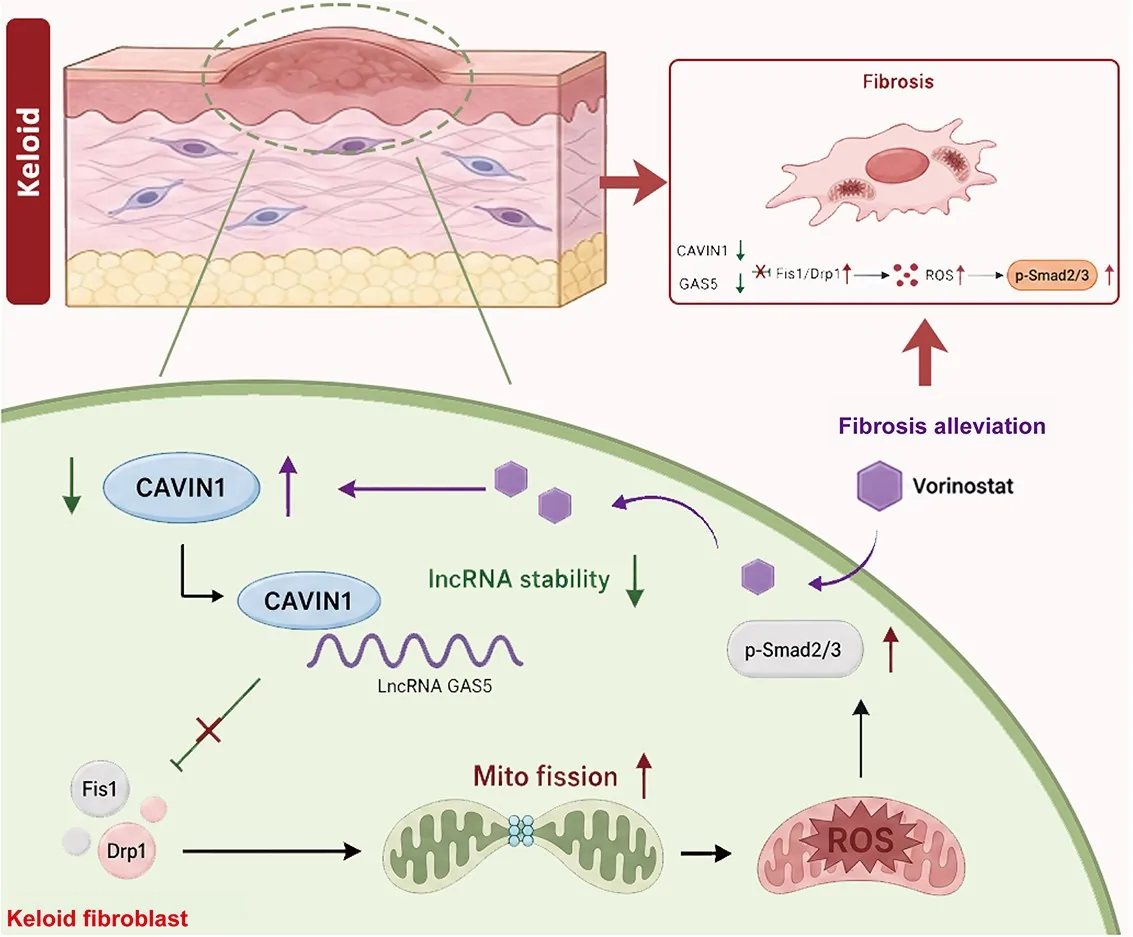
Proposed mechanism of CAVIN1 in keloids. Schematic model illustrating that CAVIN1 stabilizes the lncRNA GAS5, which in turn suppresses mitochondrial fission and subsequent ROS production. This cascade leads to the inhibition of the TGF-β/Smad signaling pathway, thereby attenuating fibroblast activation and collagen deposition. Vorinostat has been identified as a pharmacological agent that upregulates CAVIN1, thus potentiating this antifibrotic axis. ROS reactive oxygen species, *CAVIN1* caveolae-associated protein 1

The present study identified CAVIN1 as a novel antifibrotic regulator against keloid pathogenesis, demonstrating that its overexpression attenuates fibroblast activation and ECM deposition by stabilizing the long noncoding RNA GAS5. This stabilization suppressed Drp1/Fis1-mediated mitochondrial fission, reduced ROS generation, and consequently inhibited the canonical TGF-β/Smad signaling pathway (Figure 7). The resulting CAVIN1/GAS5 axis establishes a previously unrecognized link between caveolae-associated proteins and mitochondrial dynamics in dermal fibrosis, highlighting a multifaceted mechanism that concurrently targets upstream mitochondrial dysfunction and downstream profibrotic signaling.

TGF-β signaling is the predominant driver of fibrosis across organs, including the skin, with its sustained activation resulting in the phosphorylation of Smad2/3, the promotion of nuclear Smad2/3 translocation, and the upregulation of ECM genes, such as collagen and POSTN [23]. This process is amplified by a self-perpetuating inflammation–fibrosis cycle that involves inflammatory cell recruitment and persistent low-grade inflammation [24]. In keloids, aberrant TGF-β activation contributes to the resistance of fibroblasts to apoptosis and their uncontrolled proliferation. Our findings align with the established antifibrotic role of GAS5 in cardiac, hepatic, and renal fibrosis, in which it modulates pathways such as the PTEN/PI3K/Akt or Smad3–miRNA interactions [16, 25, 26]. Notably, we extended this role of GAS5 to skin fibrosis by revealing the novel suppression of mitochondrial fission by GAS5—a mechanism that is unexplored in dermal contexts but is increasingly implicated in organ-specific fibrogenesis [27].

Mitochondrial dynamics are pivotal for cellular homeostasis, with excessive fission, which is driven by Drp1 and Fis1, promoting ROS overproduction and fibrogenesis in pulmonary, renal, and hepatic models [28–30]. In keloids, mitochondrial fragmentation and dysfunction are correlated with altered bioenergetics and resistance to apoptosis [31]. Our data revealed increased expression of Drp1/Fis1 in KFs and a reduction of ROS levels via GAS5-dependent inhibition by CAVIN1, which is consistent with evidence that mitochondrial fission–ROS cascades amplify TGF-β signaling [32]. This vicious cycle—in which ROS activate latent TGF-β and mitochondrial fission sustains ROS production—underpins persistent fibrosis [33]. Notably, given the pleiotropic nature of both mitochondrial dynamics and GAS5, additional parallel pathways may also contribute to the observed antifibrotic outcomes, and the relative contributions of these mechanisms warrant further investigation.

Critically, the role of CAVIN1 complements that of related caveolar proteins; for example, reduced caveolin-1 (Cav-1) expression increases TGF-β signaling and fibrosis in keloids, suggesting that caveolar components broadly regulate mechanotransduction and ECM remodeling in dermal disorders [34, 35]. While the reduction in Drp1 and Fis1 expression strongly supports the conclusion that the CAVIN1/GAS5 axis suppresses mitochondrial fission, direct ultrastructural evidence—such as transmission electron microscopy or live-cell MitoTracker imaging data—will be valuable in future studies to fully characterize the dynamics of mitochondrial network remodeling in KFs.

The therapeutic implications of our findings are highlighted by the identification of vorinostat, an HDAC inhibitor, as a compound capable of upregulating CAVIN1 expression and mimicking the antifibrotic effects of the CAVIN1/GAS5 axis. HDAC inhibitors, such as vorinostat and its analog trichostatin A (TSA), have demonstrated antifibrotic potential in various models by modulating epigenetic regulation of profibrotic genes, reducing collagen synthesis, and normalizing aberrant gene expression in fibroblasts [36–39]. Although direct evidence in keloids is limited, upregulation of HDAC2 in keloid scars and normalization of epigenetic alterations in KFs following TSA treatment suggest that HDAC inhibitors may hold promise for keloid therapy [40, 41]. However, the translation of these findings into clinical applications requires comprehensive evaluation, including dose–response analyses, long-term safety assessments, and head-to-head comparisons with established keloid treatments, such as intralesional corticosteroid injection [42, 43]. Furthermore, targeting mitochondrial fission with Drp1 inhibitors, such as Mdivi-1, represents a complementary precision strategy to attenuate the mitochondrial fission–ROS–TGF-β cascade, as Mdivi-1 has been shown to ameliorate fibrosis in cardiac, renal, pulmonary, and hepatic models by inhibiting Drp1-mediated mitochondrial fragmentation and oxidative stress [28–30, 44, 45]. While these approaches offer theoretical advantages over broad inhibition of TGF-β, which is associated with the risk of off-target effects because of the essential roles of TGF-β in wound healing, immune regulation, and tumor suppression [**23****]**, their efficacy and safety in keloid-specific settings remain to be rigorously tested.

While our in vivo experiments confirmed that AAV-mediated CAVIN1 overexpression combined with vorinostat treatment significantly ameliorated skin fibrosis, the results of the current study primarily validated the antifibrotic efficacy at the histological level. Given that the mechanistic characterization of CAVIN1 was conducted mainly in cultured fibroblasts, its functional role within the complex in vivo microenvironment warrants further investigation. To this end, advanced technologies, such as single-cell RNA-seq and spatial transcriptomics, can be employed to dissect the cellular heterogeneity and intercellular crosstalk underlying CAVIN1-mediated antifibrotic effects to enable a comprehensive understanding of how CAVIN1 orchestrates molecular changes across diverse cell types in the skin microenvironment. These insights will deepen our understanding of the therapeutic potential of CAVIN1 and inform more precise strategies for targeting fibrotic skin disorders. Additionally, although our study revealed that CAVIN1 directly bound to and stabilized GAS5, the precise molecular determinants of this interaction—including the specific RNA-binding domains of CAVIN1 and the degradation pathways that it may antagonize—remain to be elucidated and warrant further investigation.

This study employed a bleomycin-induced mouse model of skin fibrosis, which cannot fully recapitulate all the pathophysiological features of human keloids [46]. Bleomycin-induced fibrosis is typically transient and spontaneously resolves after cessation of bleomycin injections [47], whereas keloids in humans are characterized by persistent and progressive fibrosis that does not regress spontaneously. Furthermore, the bleomycin model primarily reflects inflammation-driven fibrosis, while the pathogenesis of keloids involves additional factors, such as mechanical tension and genetic predisposition [48]. These differences may limit the direct applicability of our findings to human keloids. Nevertheless, owing to its high reproducibility and well-characterized fibrotic response, the bleomycin model remains valuable for initial mechanistic studies and therapeutic screening [49]. In future studies, we will further investigate the use of a human keloid xenograft model in immunodeficient mice. Moreover, the initial multiomics findings in this study were based on a limited sample size. In future studies, we will collect additional clinical samples to validate the value of CAVIN1 as a novel therapeutic target for keloids in larger, independent patient cohorts.

## Conclusions

In summary, the CAVIN1/GAS5/mitochondrial fission axis represents a previously unrecognized mechanistic link among the function of caveolae, mitochondrial homeostasis, and TGF-β signaling in the pathogenesis of keloids. Strategies aimed at upregulating CAVIN1 expression, stabilizing GAS5, or inhibiting mitochondrial fission thus represent innovative therapeutic avenues, with vorinostat emerging as a promising repurposable drug candidate that warrants further translational evaluation in fibrotic skin disorders.

## Supplementary Material

Supplementary_Figure_S1_caption_tkag052

Supplementary_File_1_tkag052

Supplementary_File_2_tkag052

## Data Availability

The data used to support the findings of this study are available from the corresponding author upon reasonable request.

## References

[1] Zhang M, Chen H, Qian H, Wang C. Characterization of the skin keloid microenvironment. *Cell Commun Signal* 2023;21:207. 10.1186/s12964-023-01214-0. PMC10428592 37587491

[2] Kim HJ, Kim YH. Comprehensive insights into keloid pathogenesis and advanced therapeutic strategies. *Int J Mol Sci* 2024;25:8776. 10.3390/ijms25168776. PMC11354446 39201463

[3] Bijlard E, Kouwenberg CA, Timman R, Hovius SE, Busschbach JJ, Mureau MA. Burden of keloid disease: a cross-sectional health-related quality of life assessment. *Acta Derm Venereol* 2017;97:225–9. 10.2340/00015555-2498. 27378582

[4] Yin Q, Niessen FB, Gibbs S, Lapid O, Louter JMI, Zuijlen v, et al. Intralesional corticosteroid administration in the treatment of keloids: a survey among Dutch dermatologists and plastic surgeons. *J Dermatolog Treat* 2023;34:2159308. 10.1080/09546634.2022.2159308. 36594683

[5] Leszczynski R, da Silva CA, Pinto ACPN, Kuczynski U, da Silva EM. Laser therapy for treating hypertrophic and keloid scars. *Cochrane Database Syst Rev* 2022;2022:CD011642. 10.1002/14651858.CD011642.pub2. PMC9511989 36161591

[6] Ekstein SF, Wyles SP, Moran SL, Meves A. Keloids: a review of therapeutic management. *Int J Dermatol* 2021;60:661–71. 10.1111/ijd.15159. PMC7940466 32905614

[7] Meng XM, Nikolic-Paterson DJ, Lan HY. TGF-β: the master regulator of fibrosis. *Nat Rev Nephrol* 2016;12:325–38. 10.1038/nrneph.2016.48. 27108839

[8] Hu HH, Chen DQ, Wang YN, Feng YL, Cao G, Vaziri ND, et al. New insights into TGF-β/Smad signaling in tissue fibrosis. *Chem Biol Interact* 2018;292:76–83. 10.1016/j.cbi.2018.07.008. 30017632

[9] Kelsh RM, McKeown-Longo PJ, Clark RAF. EDA fibronectin in keloids create a vicious cycle of fibrotic tumor formation. *J Invest Dermatol* 2015;135:1714–8. 10.1038/jid.2015.155. 26066891

[10] Si S, Liu H, Xu L, Zhan S. Identification of novel therapeutic targets for chronic kidney disease and kidney function by integrating multi-omics proteome with transcriptome. *Genome Med* 2024;16:84. 10.1186/s13073-024-01356-x. PMC11186236 38898508

[11] Tillu VA, Rae J, Gao Y, Ariotti N, Floetenmeyer M, Kovtun O, et al. Cavin1 intrinsically disordered domains are essential for fuzzy electrostatic interactions and caveola formation. *Nat Commun* 2021;12:931. 10.1038/s41467-021-21035-4. PMC7875971 33568658

[12] Wu Y, Lim YW, Stroud DA, Martel N, Hall TE, Lo HP, et al. Caveolae sense oxidative stress through membrane lipid peroxidation and cytosolic release of CAVIN1 to regulate NRF2. *Dev Cell* 2023;58:376–397.e4. 10.1016/j.devcel.2023.02.004. 36858041

[13] Taniguchi T, Maruyama N, Ogata T, Kasahara T, Nakanishi N, Miyagawa K, et al. PTRF/Cavin-1 deficiency causes cardiac dysfunction accompanied by cardiomyocyte hypertrophy and cardiac fibrosis. *PLoS One* 2016;11:e0162513. 10.1371/journal.pone.0162513. PMC5017623 27612189

[14] Zhou Y, Chen B. GAS5-mediated regulation of cell signaling (review). *Mol Med Rep* 2020;22:3049–56. 10.3892/mmr.2020.11435. PMC7453608 32945519

[15] Tu B, Song K, Zhou Y, Sun H, Liu Z, Lin L, et al. METTL3 boosts mitochondrial fission and induces cardiac fibrosis by enhancing LncRNA GAS5 methylation. *Pharmacol Res* 2023;194:106840. 10.1016/j.phrs.2023.106840. 37379961

[16] Zhang YY, Tan RZ, Yu Y, Niu YY, Yu C. LncRNA GAS5 protects against TGF-β-induced renal fibrosis via the Smad3/miRNA-142-5p axis. *Am J Physiol Renal Physiol* 2021;321:F517–26. 10.1152/ajprenal.00085.2021. 34486400

[17] Dong Z, Li S, Wang X, Si L, Ma R, Bao L, et al. lncRNA GAS5 restrains CCl4-induced hepatic fibrosis by targeting miR-23a through the PTEN/PI3K/Akt signaling pathway. *Am J Physiol Gastrointest Liver Physiol* 2019;316:G539–50. 10.1152/ajpgi.00249.2018. 30735452

[18] Patra S, Praharaj PP, Klionsky DJ, Bhutia SK. Vorinostat in autophagic cell death: a critical insight into autophagy-mediated, −associated and -dependent cell death for cancer prevention. *Drug Discov Today* 2022;27:269–79. 10.1016/j.drudis.2021.08.004. PMC8714665 34400351

[19] Yang H, Xu H, Wang Z, Li X, Wang P, Cao X, et al. Analysis of miR-203a-3p/SOCS3-mediated induction of M2 macrophage polarization to promote diabetic wound healing based on epidermal stem cell-derived exosomes. *Diabetes Res Clin Pract* 2023;197:110573. 10.1016/j.diabres.2023.110573. 36764461

[20] Yang H, Lv D, Li X, Chen Y, Xu H, Wu H, et al. Comprehensive analysis of keloid super-enhancer networks reveals FOXP1-mediated anti-senescence mechanisms in fibrosis. *Cell Mol Biol Lett* 2025;30:88. 10.1186/s11658-025-00763-1. PMC12288304 40702440

[21] Yi K, Cui X, Liu X, Wang Y, Zhao J, Yang S, et al. PTRF/Cavin-1 as a novel RNA-binding protein expedites the NF-κB/PD-L1 Axis by stabilizing lncRNA NEAT1, contributing to tumorigenesis and immune evasion in glioblastoma. *Front Immunol* 2022;12:802795. 10.3389/fimmu.2021.802795. PMC8778801 35069587

[22] Yu T, Wang L, Zhang L, Deuster PA. Mitochondrial fission as a therapeutic target for metabolic diseases: insights into antioxidant strategies. *Antioxidants (Basel)* 2023;12:1163. 10.3390/antiox12061163. PMC10295595 37371893

[23] Zhang T, Wang XF, Wang ZC, Lou D, Fang QQ, Hu YY, et al. Current potential therapeutic strategies targeting the TGF-β/Smad signaling pathway to attenuate keloid and hypertrophic scar formation. *Biomed Pharmacother* 2020;129:110287. 10.1016/j.biopha.2020.110287. 32540643

[24] Wynn TA . Cellular and molecular mechanisms of fibrosis. *J Pathol* 2008;214:199–210. 10.1002/path.2277. PMC2693329 18161745

[25] Tao H, Zhang JG, Qin RH, Dai C, Shi P, Yang JJ, et al. LncRNA GAS5 controls cardiac fibroblast activation and fibrosis by targeting miR-21 via PTEN/MMP-2 signaling pathway. *Toxicology.* 2017;386:11–8. 10.1016/j.tox.2017.05.007. 28526319

[26] Yu F, Zheng J, Mao Y, Dong PH, Lu ZQ, Li GJ, et al. Long non-coding RNA growth arrest-specific transcript 5 (GAS5) inhibits liver fibrogenesis through a mechanism of competing endogenous RNA. *J Biol Chem* 2015;290:28286–98. 10.1074/jbc.M115.683813. PMC4653684 26446789

[27] Xiang Z, Liqing Y, Qingqing Y, Qiang H, Hongbo C. Retard or exacerbate: role of long non-coding RNA growth arrest-specific 5 in the fibrosis. *Cytokine Growth Factor Rev* 2022;67:89–104. 10.1016/j.cytogfr.2022.06.001. 35753990

[28] Tong Z, Du X, Zhou Y, Jing FX, Ma JP, Feng YY, et al. Drp1-mediated mitochondrial fission promotes pulmonary fibrosis progression through the regulation of lipid metabolic reprogramming by ROS/HIF-1α. *Cell Signal* 2024;117:111075. 10.1016/j.cellsig.2024.111075. 38311302

[29] Wang Y, Lu M, Xiong L, Fan JJ, Zhou Y, Li HY, et al. Drp1-mediated mitochondrial fission promotes renal fibroblast activation and fibrogenesis. *Cell Death Dis* 2020;11:29. 10.1038/s41419-019-2218-5. PMC6965618 31949126

[30] Wang J, Yang Y, Sun F, Luo Y, Yang Y, Li J. et al. ALKBH5 attenuates mitochondrial fission and ameliorates liver fibrosis by reducing Drp1 methylation. *Pharmacol Res* 2023;187:106608. 10.1016/j.phrs.2022.106608. 36566000

[31] Chen B, Yu D, Qin Z, Zhao R, Li Y, Li Q, et al. Mitochondrial dysfunctions of keloid fibroblasts and it’s effects on cell metabolic functions. *Zhonghua Zheng Xing Wai Ke Za Zhi* 2016;32:359–64. 10.3760/cma.j.issn.1009-4598.2016.05.010. 30066994

[32] Jain M, Rivera S, Monclus EA, Synenki L, Zirk A, Eisenbart J, et al. Mitochondrial reactive oxygen species regulate transforming growth factor-β signaling. *J Biol Chem* 2013;288:770–7. 10.1074/jbc.M112.431973. PMC3543026 23204521

[33] Liu RM, Desai LP. Reciprocal regulation of TGF-β and reactive oxygen species: a perverse cycle for fibrosis. *Redox Biol* 2015;6:565–77. 10.1016/j.redox.2015.09.009. PMC4625010 26496488

[34] Zhang GY, Yu Q, Cheng T, Liao T, Nie CL, Wang AY, et al. Role of caveolin-1 in the pathogenesis of tissue fibrosis by keloid-derived fibroblasts in vitro. *Br J Dermatol* 2011;164:623–7. 10.1111/j.1365-2133.2010.10111.x. 21375514

[35] Takamura N, Yamaguchi Y. Involvement of caveolin-1 in skin diseases. *Front Immunol* 2022;13:1035451. 10.3389/fimmu.2022.1035451. PMC9748611 36532050

[36] Yoon S, Kang G, Eom GH. HDAC inhibitors: therapeutic potential in fibrosis-associated human diseases. *Int J Mol Sci* 2019;20:1329. 10.3390/ijms20061329. PMC6471162 30884785

[37] Lyu X, Hu M, Peng J, Zhang X, Sanders YY. HDAC inhibitors as antifibrotic drugs in cardiac and pulmonary fibrosis. *Ther Adv Chronic Dis* 2019;10:2040622319862697. 10.1177/2040622319862697. PMC6643173 31367296

[38] Sanders YY, Hagood JS, Liu H, Zhang W, Ambalavanan N, Thannickal VJ. Histone deacetylase inhibition promotes fibroblast apoptosis and ameliorates pulmonary fibrosis in mice. *Eur Respir J* 2014;43:1448–58. 10.1183/09031936.00095113. 24603818

[39] Diao JS, Xia WS, Yi CG, Wang YM, Li B, Xia W, et al. Trichostatin a inhibits collagen synthesis and induces apoptosis in keloid fibroblasts. *Arch Dermatol Res* 2011;303:573–80. 10.1007/s00403-011-1140-1. 21400246

[40] Fitzgerald O'Connor EJ, Badshah II, Addae LY, Kundasamy P, Thanabalasingam S, Abioye D, et al. Histone deacetylase 2 is upregulated in normal and keloid scars. *J Invest Dermatol* 2012;132:1293–6. 10.1038/jid.2011.432. 22205303

[41] Diao JS, Xia WS, Yi CG, Yang Y, Zhang X, Xia W, et al. Histone deacetylase inhibitor reduces hypertrophic scarring in a rabbit ear model. *Plast Reconstr Surg* 2013;132:61e–9. 10.1097/PRS.0b013e318290f698. 23806955

[42] Yin Q, Wolkerstorfer A, Lapid O, Qayumi K, Alam M, Al-Niaimi F, et al. KECORT study: an international e-Delphi study on the treatment of KEloids using Intralesional CORTicosteroids in clinical practice. *Am J Clin Dermatol* 2024;25:1009–17. 10.1007/s40257-024-00888-7. PMC11511692 39298112

[43] Zhu K, Song Q, Pan L, Xiong F. Efficacy and safety of glucocorticoid-based therapies in the management of keloids: a systematic review and meta-analysis of clinical outcomes. *Front Med (Lausanne)* 2026;12:1749329. 10.3389/fmed.2025.1749329. PMC12832321 41601744

[44] Ding J, Zhang Z, Li S, Wang W, Du TY, Fang Q, et al. Mdivi-1 alleviates cardiac fibrosis post myocardial infarction at infarcted border zone, possibly via inhibition of Drp1-activated mitochondrial fission and oxidative stress. *Arch Biochem Biophys* 2022;718:109147. 10.1016/j.abb.2022.109147. 35143784

[45] Gegg ME, Cooper JM, Chau KY, Rojo M, Schapira AH, Taanman JW. Mitofusin 1 and mitofusin 2 are ubiquitinated in a PINK1/parkin-dependent manner upon induction of mitophagy. *Hum Mol Genet* 2010;19:4861–70. 10.1093/hmg/ddq419. PMC3583518 20871098

[46] Limandjaja GC, Niessen FB, Scheper RJ, Gibbs S. The keloid disorder: heterogeneity, histopathology, mechanisms and models. *Front Cell Dev Biol* 2020;8:360. 10.3389/fcell.2020.00360. PMC7264387 32528951

[47] Tan Q, Link PA, Meridew JA, Pham TX, Caporarello N, Ligresti G, et al. Spontaneous lung fibrosis resolution reveals novel antifibrotic regulators. *Am J Respir Cell Mol Biol* 2021;64:453–64. 10.1165/rcmb.2020-0396OC. PMC8008802 33493091

[48] Tan S, Khumalo N, Bayat A. Understanding keloid pathobiology from a quasi-neoplastic perspective: less of a scar and more of a chronic inflammatory disease with cancer-like tendencies. *Front Immunol* 2019;10:1810. 10.3389/fimmu.2019.01810. PMC6692789 31440236

[49] Lee YI, Shim JE, Kim J, Lee WJ, Kim JW, Nam KH, et al. WNT5A drives interleukin-6-dependent epithelial-mesenchymal transition via the JAK/STAT pathway in keloid pathogenesis. *Burns. Trauma* 2022;10:tkac023. 10.1093/burnst/tkac023. PMC9547497 36225328

