# Supplementary material for: A novel CAVIN1/GAS5 axis suppresses skin fibrosis through mitochondrial fission-mediated attenuation of the TGF-β/Smad pathway
Source: Burns Trauma. 2026 Aug 10;14:tkag052. doi: 10.1093/burnst/tkag052 (PMC13592680; doi:10.1093/burnst/tkag052)
Supplement: Supplementary file 1 [file supplementary_figure_s1_caption_tkag052.docx]

**Figure S1. CAVIN1 is a druggable target for ameliorating skin fibrosis in vivo**

(a) Results of the western blot analysis of CAVIN1, Fis1, Drp1, Smad2/3, and p-Smad2/3 protein expression in skin tissues from the indicated groups (n=3). (b) Results of the quantitative analysis of GAS5 expression in skin tissues from the indicated groups (n=3). (c) Results of the western blot analysis of CAVIN1, Fis1, Drp1, Smad2/3, and p-Smad2/3 protein expression in skin tissues after vorinostat treatment (n=3). (d) Results of the quantitative analysis of GAS5 expression in skin tissues after vorinostat treatment (n=3). **p < 0.01. adeno-associated virus (AAV), AAV-control (AAV-CTR), phosphorylated Smad2/3 (p-Smad2/3).
