# Supplementary material for: A novel CAVIN1/GAS5 axis suppresses skin fibrosis through mitochondrial fission-mediated attenuation of the TGF-β/Smad pathway
Source: Burns Trauma. 2026 Aug 10;14:tkag052. doi: 10.1093/burnst/tkag052 (PMC13592680; doi:10.1093/burnst/tkag052)
Supplement: Supplementary file 2 [file supplementary_file_2_tkag052.docx]

**Supplementary File 2: Data of sequences for RT-qPCR**

| **Gene** | **Sequence 5’-3’** |
| --- | --- |
| CAVIN1 | Forward: GAGGACCCCACGCTCTATATT  Reverse: CCCCGATGATTTTGTCCAGGA |
| GAS5 | Forward: CAGTGTGGCTCTGGATAGCA  Reverse: TTAAGCTGGTCCAGGCAAGT |
| Drp1 | Forward: CCAGTGAAGCCGGTCAATCT  Reverse: GCTGCCGTGACAGAAAACAC |
| Fis1 | Forward: CTGAACGAGCTGGTGTCTGT  Reverse: GAACAGGGAAAGGACAGCGA |
| GAPDH | Forward: ACAACTTTGGTATCGTGGAAGG  Reverse: GCCATCACGCCACAGTTTC |
| CAVIN1-M | Forward: AGGATGTCACGCTCCATATCG  Reverse: GTTCACCTGGTCCGACTTGA |
| GAPDH-M | Forward: AATGGATTTGGACGCATTGGT  Reverse: TTTGCACTGGTACGTGTTGAT |
| GAS5-M | Forward: GTGAGGTATGGTGCTGGGTG  Reverse: GCCAATGGCTTGAGTTAGGC |
